# Self-Health Monitoring by Smart Devices and Ontology Technology for Older Adults With Uncontrolled Hypertension: Quasi-Experimental Study

**DOI:** 10.2196/73386

**Published:** 2025-10-14

**Authors:** Sutteeporn Moolsart, Khajitpan M Kritpolviman

**Affiliations:** 1 School of Nursing, Sukhothai Thammathirat Open University, 9/9 Mhu 9, Bang Phut Sub-district, Pak Kret District, Nonthaburi, 11120, Thailand, 66 0860318420; 2 School of Science and Technology, Sukhothai Thammathirat Open University, Pak Kret District, Nonthaburi, Thailand

**Keywords:** Thailand, self-health monitoring, smart devices, ontology technology, older adults, uncontrolled hypertension

## Abstract

**Background:**

Hypertension is a prevalent concern among older adults, often leading to complex cardiovascular complications when uncontrolled. Telenursing technology facilitates self-management, and the integration of domain-specific ontology allows real-time interpretation of behavioral and biometric data to provide personalized recommendations, enhancing patient engagement and self-care.

**Objective:**

This study aimed to examine the within-group and between-group effects of self-health monitoring using smart devices combined with ontology technology on hypertension-controlling behavior and mean arterial pressure among older adults with uncontrolled hypertension.

**Methods:**

The quasi-experimental design was conducted with 91 older adults in Bangkok, Thailand (46 experimental and 45 comparison participants). Participants in the experimental group used the “HT GeriCare@STOU” app on smartphones, linked to smartwatches for blood pressure monitoring, step count, and sleep pattern, with telenursing support via video calls. Data on hypertension-controlling behavior were collected using a validated questionnaire (Cronbach *α*=0.83; content validity index=0.98). Descriptive statistics and *t* tests were used to analyze within-group and between-group differences.

**Results:**

Within-group analysis revealed that experimental participants showed improved hypertension-controlling behavior and reduced mean arterial pressure after the program. Between-group comparisons indicated that mean arterial pressure in the experimental group was significantly lower than in the comparison group (*P*<.05), although hypertension-controlling behavior did not differ significantly between groups. Older adult participants and nurses reported high satisfaction, noting that real-time feedback increased awareness of blood pressure and motivated independent health behavior adjustments.

**Conclusions:**

Self-health monitoring using smart devices integrated with ontology technology effectively improved physiological outcomes and supported self-management in older adults with uncontrolled hypertension. The ontology framework enabled personalized, real-time decision support, highlighting its novelty, and potential to enhance nursing practice. Future studies with larger samples and longer follow-up are recommended to further evaluate the intervention’s effectiveness and scalability.

## Introduction

### Background

Hypertension is a major public health challenge worldwide, with its prevalence continuing to rise, particularly among older adults. In Thailand, the age-standardized prevalence of hypertension among older adults was estimated at 60% in 2019‐2020. Alarmingly, 77.3% of hypertensive patients nationwide had uncontrolled blood pressure (>140/90 mm Hg), reflecting a critical gap in disease management [1].

Bangkok, which officially became an aging society between 2020 and 2022, saw its older adult population increase from 20.40% to 21.6%. In 2021 alone, 1.23 million older adults were living in the city, 20.60% of whom had hypertension [2]. During the same period, the proportion of patients with uncontrolled hypertension rose from 40.62% in 2020 to 60.55% in 2022, surpassing the city’s target of keeping this figure below 50% [3]. In 2022, 102,471 hypertensive patients received care at 69 public health centers [3], and more than half were estimated to have inadequately controlled blood pressure. These trends underscore the urgent need for targeted blood pressure management strategies for older adults, aiming for systolic blood pressure (SBP) <140 mm Hg or diastolic blood pressure (DBP) <90 mm Hg to prevent complications.

The Ministry of Public Health has implemented measures to improve health behaviors in at-risk groups and patients with chronic diseases, such as reducing sugar, fat, and salt intake, promoting smoke- and alcohol-free habits, and encouraging home blood pressure monitoring. Hypertension management in Bangkok communities follows these guidelines [4]: (1) annual follow-ups for registered patients, (2) education and behavioral modification on at least 3 topics per year, (3) a referral system to public health centers or hospitals for patients with complications, and (4) monitoring blood pressure, with monthly home visits for patients with uncontrolled issues. Empowering hypertensive patients, especially older adults, to manage their health ensures sustainable control and may reduce health care costs. However, changing health behaviors in older adults is challenging and requires significant time, resources, and consistent effort.

A study found that older adults with hypertension often felt frustrated with management, as their blood pressure fluctuated despite their self-care efforts, leading to decreased motivation [5]. Key barriers to self-care include motivation and age. The 2022 evaluation of noncommunicable disease services showed that efforts to modify health behaviors in diabetes and hypertension patients were unclear, inconsistent, and not personalized to individual lifestyles [6]. This lack of clarity and consistency contributed to the low motivation among at-risk individuals and patients to adopt healthier behaviors.

To prevent complications in patients with chronic diseases, the Ministry of Public Health has proposed guidelines to enhance health literacy and self-awareness (Know Your Number) among normal, at-risk, suspected, and patient groups. This included communicating risks to encourage awareness and behavior modification in both diabetes and hypertension patients. Given the challenges faced by older adults and the health care system, it was clear that empowering adults and older adult patients with hypertension to independently seek knowledge, make informed decisions, and manage their behaviors was crucial for addressing daily life challenges related to hypertension.

A systematic review of self-management found that, over 12 months, it could reduce SBP by 3.2 mm Hg [7,8]. Another review of hypertension intervention activities from 2008 to 2018 [9] showed that combining health education, counseling, and management effectively reduced blood pressure, lowering systolic by −5.34 mm Hg and diastolic by −3.23 mm Hg. Tucker et al [8] found that self-monitoring alone was not sufficient to reduce blood pressure; it should be combined with cointerventions and supported by close monitoring, including systematic medication adjustments in collaboration with doctors, pharmacists, and patients, along with health education or counseling for lifestyle changes.

Previous studies have shown that hypertension management was typically carried out by doctors, nurses, or community health volunteers, which could lead to patients becoming overly dependent on the health care system. However, with about 90% of urban Thai people using electronic media, studies indicated that older adults had a high level of technology acceptance. Moolsart and Kritpolviman [10] found that 76.73% of older adult patients with uncontrolled hypertension receiving care at public health centers in Bangkok owned smartphones, with LINE (LY Corporation) being the most commonly used app. Furthermore, older adults showed a moderate interest in using information technology for managing their health conditions.

The prevention and treatment of high blood pressure could be greatly aided by mobile health (mHealth) technologies [11]. However, technology had not been widely used for treating hypertension in older adults, and there was still limited research in this area. Therefore, developing electronic media for self-monitoring health conditions in older adults with hypertension was an effective way to provide knowledge tailored to urban lifestyles. Allowing older adults to continuously assess their health and manage behaviors related to diet, exercise, stress, and medication adherence could help control their condition, reducing the need for medical treatment and hospital visits. Chaipattanamethi [12] developed a multimedia food system on dietary approaches to stop hypertension (DASH) for older adults, which was a lesson installed on portable personal computers (notebooks). When tested with older adults, user satisfaction was found to be high overall.

### Research Rationale and Aim

This study aimed to assess the effectiveness of technology-based interventions for monitoring the health conditions of older adults. Specifically, it focused on the impact of self-health monitoring using smart devices combined with ontology technology on hypertension-controlling behavior and mean arterial pressure (MAP) among older adults with uncontrolled hypertension.

Ontology refers to a formal representation of knowledge comprising a set of concepts and their interrelationships, designed to be shared and reused across systems. In health informatics, ontology enables the structured organization of domain-specific knowledge, supporting semantic understanding, automated reasoning, and intelligent information retrieval [13-15]. In this study, ontology technology was used to construct a health knowledge base tailored to older adults with hypertension. This ontology-based system served as the foundation for semantic web technologies, enhancing the accuracy and efficiency of health information access through interconnected and user-centered data services.

To operationalize this approach, the intervention integrated the HT GeriCare@STOU mobile app [10], smartwatch-based blood pressure monitoring, training in health knowledge and digital literacy, telenursing via LINE for home-based care, and professional-led online knowledge sharing. These components were designed to promote self-regulation: self-observation through real-time monitoring of blood pressure and physical activity via smartwatch; self-judgment through personalized feedback and goal tracking in the mobile app; and self-reaction through behavioral prompts, telenursing support, and health education. It was hypothesized that older adults who previously struggled to control their hypertension would improve their self-management skills, thereby reducing complications and enhancing their quality of life.

## Methods

### Study Design

A 2-group pretest-posttest design was used in this quasi-experimental investigation. The study, which lasted 12 weeks, examined the effects of ontology technology and smart device-based self-health monitoring on hypertension-controlling behavior and MAP in older adults with uncontrolled hypertension.

### Sample and Setting

The sample group consisted of older adults aged 60‐75 years who had been diagnosed with essential hypertension and exhibited uncontrolled blood pressure. All participants resided in Bangkok.

To organize the sampling framework, Bangkok’s 69 public health facilities were grouped into 6 administrative zones. Two of these zones were selected by matched community clusters to form experimental and comparison groups. Authorization to conduct the study was obtained from 13 public health facilities within these zones. Among these, 8 facilities were assigned to either the experimental or comparison group based on their geographic location to minimize cross-contamination. The remaining 4 facilities had a small number of eligible participants; one of these was selected for piloting the research instruments. Although randomization was not applied, this quasi-experimental design used geographical allocation and comparable facility profiles to reduce potential selection bias.

The sample size was calculated using the G*power program (Heine University of Düsseldorf) applying the statistical test means: the variation of 2 independent means (2 groups). The effect size was examined and computed as 0.7, representing a medium effect size (as no research contrasts with this study, this is an approximation). Using an alpha value at .05 and a power of .95, the sample size calculation program determined 45 people per group. However, in this study, to prevent sample loss, an additional 15% was added to the sample size. Therefore, the sample size in both the experimental and control groups was increased to 52 people each, totaling 104 people.

Due to ethical constraints, random sampling from lists of patients with uncontrolled hypertension was not feasible, as patient identities could not be disclosed by the participating health facilities. Therefore, a voluntary enrollment approach was used. Older adults who met the inclusion criteria were invited to participate through health facility announcements and outreach by local health personnel. Participants were then screened and selected based on eligibility criteria.

**Figure 1.**
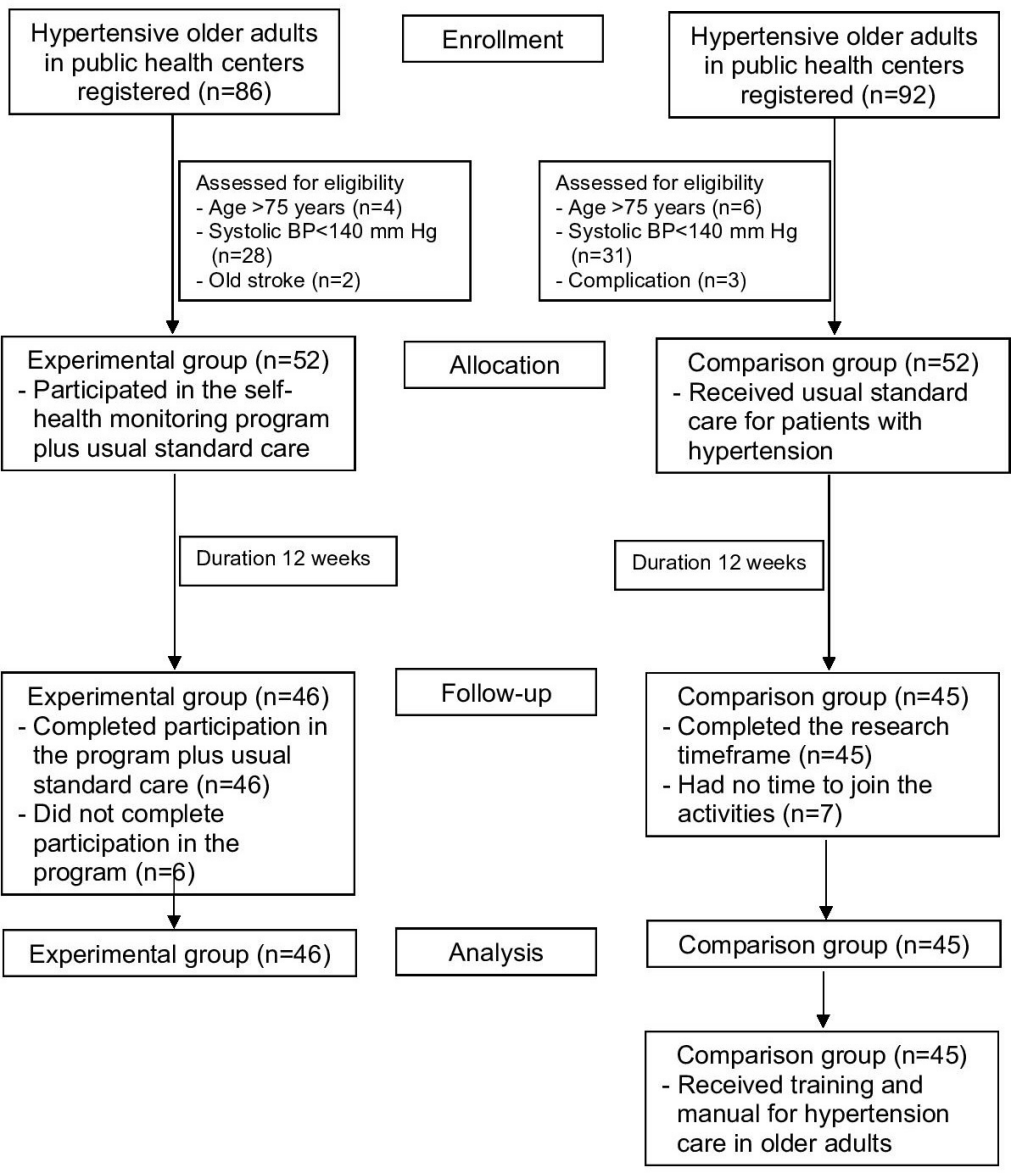
A Consolidated Standards of Reporting Trials (CONSORT) flow diagram of selecting the participants in this study. BP: blood pressure.

The inclusion criteria were older adults aged 60‐75 years who were hypertensive with SBP levels exceeding 140‐180 mm Hg or DBP levels exceeding 90‐109 mm Hg. They did not have any comorbidities or complications related to hypertension, such as diabetes, cardiovascular diseases, kidney diseases, and eye diseases. They could read and write, had normal cognitive function, and were not depressed, which would be screened using the Thai version of the Mini-Cog screening tool [16] and the 2-question (2Q) depression assessment. Older adults or their caregivers using smartphones were recruited. Exclusion criteria included withdrawal from the program, problems, or a major sickness that prevented participation, as well as participation in the program for fewer than 12 weeks in a row. A total of 86 and 92 older adults initially volunteered for the experimental and comparison groups, respectively. Following eligibility screening based on the inclusion criteria, 52 participants were included in each group (Figure 1). Both groups of participants joined the program and engaged in the following activities: (1) the experimental group received the self-health monitoring program and usual standard care for 12 weeks; (2) the comparison group received care services according to the standards of the public health service centers, and at the end of the 12-week trial, they underwent teaching and a hypertension manual to develop their self-health management skills. After 12 weeks, 46 participants (88.46%) in the experimental group and 45 participants (86.54%) in the comparison group completed the study, as shown in Figure 1.

To minimize potential confounding factors, the study applied strict inclusion criteria. All participants were diagnosed with essential hypertension and had been receiving similar classes of antihypertensive medications with stable regimens for at least four weeks before enrollment. Participants with significant comorbidities such as diabetes, cardiovascular diseases, renal insufficiency, or cognitive impairment were not recruited to reduce variability. Moreover, the study required that all participants had a designated caregiver who could assist with self-monitoring activities and attend training sessions. This ensured consistency in caregiver engagement across both intervention and comparison groups, reducing its potential confounding effect.

### Instruments

**Figure 2.**
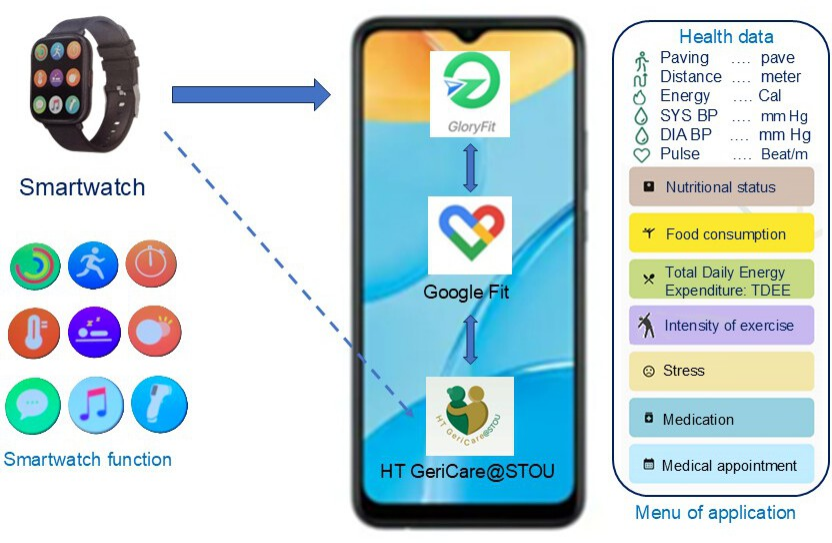
The linkage between the smartwatch and the HT GeriCare@STOU app on the smartphone and the menus.

The research implementation tool was a self-health monitoring program designed by researchers for older adults who had difficulty managing their hypertension. The program was grounded in Bandura’s [17] self-regulation theory and lasted for 12 weeks. The intervention included a smartwatch capable of measuring key life indicators such as blood pressure, heart rate, body temperature, blood oxygen levels, daily steps, and sleep patterns. The smartwatch data were automatically transmitted to a mobile app called “HT GeriCare@STOU” developed by the research team [10] as shown in Figure 2.

The app was developed based on prior formative work, including needs assessment and usability testing, to ensure it met user needs and facilitated engagement. Built as an ontological technology, the mobile app was developed based on a domain-specific ontology for hypertension management. This ontology-based system allowed the integration and semantic interpretation of user data, including dietary intake, physical activity (total daily energy expenditure), stress levels, and biometric information. The ontology enabled the app to perform basic rule-based reasoning—categorizing blood pressure readings as high, normal, or low— and generating personalized feedback or alerts accordingly. It also provided suggestions for stress reduction, dietary modification, and exercise intensity based on predefined knowledge rules and health guidelines. These data were made accessible to nurses through a linked dashboard to support individualized planning before home-based care by LINE, thereby enhancing both patient self-regulation and professional support. For example, when a participant’s blood pressure was ≥140/90 mm Hg, the system inferred high blood pressure and generated the message: “*Please recheck your medication adherence, reduce risk factors, sleep 6‐8 hours, practice meditation, among other recommendations.*”

Cognitive function (Mini-Cog) screening tool owned by the Institute of Geriatric Medicine was permitted to be used. Mini-Cog is a brief cognitive screening tool used to detect dementia or cognitive impairment in older adults. It consists of a 3-item recall test and a clock-drawing test. Scoring ranges from 0 to 5, with recall of each word scoring 1 point and the clock drawing scored 0 or 2 points. A total score below 3 indicates possible cognitive impairment. Mini-Cog is widely used due to its simplicity, speed (approximately 3 minutes), and good sensitivity and specificity for early detection of cognitive decline. Depression was assessed by asking two questions: (1) During the past 2 weeks, have you often felt down, depressed, or hopeless? And (2) During the past 2 weeks, have you often had little interest or pleasure in doing things? The 2Q depression screening tool is a freely accessible instrument that has been recommended for routine use by the Department of Mental Health, Ministry of Public Health, Thailand. As a public domain tool, it does not require prior permission for application in research or clinical practice. A positive response to either question indicated the need for further evaluation. If any cognitive impairment and depression were detected using the Mini-Cog and 2Q, the participant would be referred to the nurse at the local health service center for further evaluation and care. Such individuals would not be recruited from the study sample.

The data collection tools developed by researchers included a hypertension-controlling behavior questionnaire. The questionnaire consisted of two sections: (1) general demographic and health information (24 items), and (2) 39 items on hypertension-controlling behaviors, organized into 4 domains: food consumption, exercise, stress management, and medication adherence. Each item was rated on a 5-point Likert scale [18], ranging from 1 (never practiced) to 5 (regularly practiced). Behavioral scores were interpreted as follows: least practiced (1.00‐1.50), somewhat practiced (1.51‐2.50), moderately practiced (2.51‐3.50), very practiced (3.51‐4.50), and most practiced (4.51‐5.00).

The questionnaire items were developed based on a review of relevant literature, expert consultation, and alignment with national hypertension management guidelines. Sample items included: “*I reduce salt in my meals*” (food consumption domain), “*I exercise at least 30 minutes per day*” (exercise domain), and “*I take prescribed antihypertensive medication without missing doses*” (medication adherence domain).

Content validity was established through evaluation by 5 experts in community nursing, geriatric care, and behavioral science, yielding a content validity index of 0.98. A pilot test was conducted with 30 older adults who met the inclusion criteria but were not participants in the main study. The questionnaire demonstrated good internal consistency, with a Cronbach α coefficient of 0.83. Although factor analysis was not performed due to sample size limitations, the items were developed using a conceptually grounded framework and were validated through both expert review and pilot testing.

Blood pressure was measured using 3 digital automatic blood pressure monitors. All devices were calibrated and validated to ensure consistency across the three units and accuracy against the standard device used at the primary health care center.

### Interventions

Bandura’s self-regulation theory [17] believes that individuals can do something to control their thoughts, feelings, and actions based on intention and desire through practice and self-development. Self-regulation consists of three processes. (1) Self-observation is a process where individuals evaluate themselves, set goals, and monitor their performance outcomes to create motivation for changing behaviors to achieve desired goals. (2) The judgment process is comparing self-evaluation results with personal goals or standards, considering the desired outcomes and one’s own limitations. Learning and correcting lead to decisions in setting new directions that are suitable for oneself and close to the standards. (3) Self-reaction is the response to the evaluation of one’s behavior from the judgment process. If a person successfully performs a behavior according to the set goals, they will show a positive reaction toward themselves or reward themselves. But if the person performs the behavior below the target, they will show a negative reaction toward themselves, punish themselves, or not react toward themselves.

**Figure 3.**
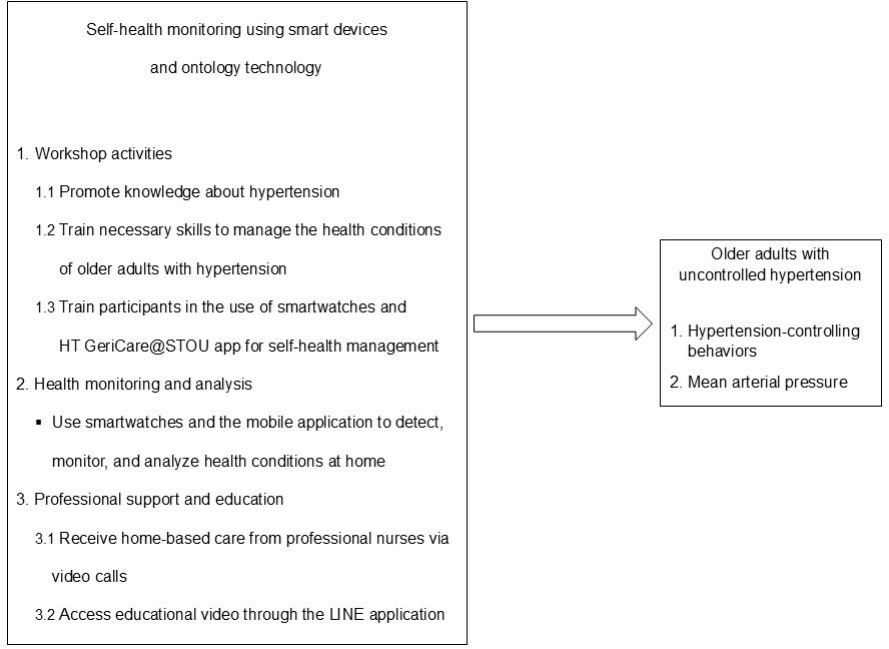
The activities and outcomes of a self-health monitoring program using smart devices combined with ontology technology.

Participants in the experimental group were able to perform self-evaluations using smartwatches to measure blood pressure, daily step count, sleep pattern, and heart rate during exercise. These data would be sent to an app “HT GeriCare@STOU” on their mobile phone. By planning previous care through online home-based care, nurses were able to identify this data. The duration of the program was 12 weeks. The first week of the program began with technological training and an invitation for nurses and the older adult to download the app to their phones. A handbook on how to use the smartwatch and its apps, as well as information on how to manage hypertension, was then given to the older adults, who also spent an hour learning and sharing their knowledge. Participants were instructed to wear the smartwatch throughout the day and night, except during bathing for the duration of the 12-week program to monitor their blood pressure, pulse, walking steps, and sleeping hours, and setting goals for hypertension control in terms of energy requirements, exercise, weight, and body mass index, as shown in Figure 3. Each morning, they measured their blood pressure, recorded body weight weekly, and logged dietary intake on 3 designated days per week. Nurses reviewed participants’ data weekly to guide personalized home-based care through LINE phone conversations and shared their knowledge and videos online throughout the third, sixth, and tenth weeks of telenursing. The research team also conducted biweekly LINE follow-ups during the first month and monthly follow-ups during months 2 and 3 to identify usage barriers and provide technical support. If participants missed recordings or encountered technical difficulties with the devices or app, the research team followed up by phone or in person to provide support and ensure protocol adherence. In the final week, the nurses and older individuals were asked to provide feedback on the program and to summarize their experiences.

### Data Collection

The data gathering and program execution occurred from June 15 to September 7, 2024. On each data collection, study assistants measured participants’ blood pressure and administered structured questionnaires regarding hypertension-controlling behaviors. The assistants confirmed that all older adults had completed the questionnaires themselves.

Participants in the experimental group received a 12-week self-monitoring program in addition to standard care. Older adult participants and their caregivers received a set of refreshments and lunch per person, as well as a fixed travel allowance. In addition, participants were provided with a smartwatch on loan for a 12-week period, accompanied by a user manual to support its use throughout the study. Adherence to the smartwatch and app usage was monitored via both automated backend data and manually recorded entries. Weekly data, including body weight, dietary intake, blood pressure, and perceived stress, were to be input regularly by participants using the app, following a standardized handout. In cases of difficulty, participants, caregivers, or community nurses could contact the research team through a dedicated LINE messaging group. Participants generally adhered to the intervention protocol, completing scheduled measurements and logs as instructed. This active monitoring and support contributed to high intervention fidelity, supporting the validity of the observed physiological and behavioral outcomes.

The comparison group received standard care only. The research assistant scheduled follow-up appointments with participants in this group for reassessment 12 weeks later, which included completing the same hypertension-controlling behavior questionnaires and measuring blood pressure. After completing the final data collection, participants in the comparison group were provided with basic education training on key topics related to healthy eating, physical activity, stress management, and medication adherence. This training was delivered after all outcome assessments were completed and was not part of the intervention program.

### Data Analysis

The computer app was used to analyze the data. In order to assess general information, descriptive statistics such as frequency, percentage, mean, and SD were used. Using the Kolmogorov-Smirnov test, the pre-experimental data were examined for normality under the *t* test (1-tailed) statistical assumptions.

Given the study’s quasi-experimental design with 2 groups (intervention and comparison) and 2 time points (pre- and postintervention), paired *t* tests were used to compare within-group changes, and independent *t* tests were used to compare between-group differences.

Although repeated measures ANOVA or mixed-effects models are often applied in studies with longitudinal data, these methods were not used due to the limited sample size, unequal group sizes, and the fact that only 2 time points were assessed. The statistical assumptions for using *t* tests, including normality and homogeneity of variance, were tested and found to be acceptable. Therefore, the *t* test was considered the most appropriate and interpretable method for this study. All statistical analyses were performed using SPSS (version 27; IBM Corporation). A *P* value of <.05 was considered statistically significant.

### Ethical Considerations

The research was approved by the School of Nursing Ethical Committee, Sukhothai Thammathirat Open University (NS 1/2566; Jan 12, 2023) and the Bangkok Research Ethics Committee (E007he/67; June 4, 2024). The trial was registered with the Thai Clinical Trials Registry (TCTR20250110003). All participants were informed about the study objectives, procedures, confidentiality, potential risks, and benefits and provided written informed consent prior to participation. Participation was voluntary, and participants could withdraw at any time without any impact on their health care. Data were kept confidential and used exclusively for research purposes.

## Results

### Participant Characteristics

The demographic characteristics of participants in both the experimental and comparison groups were similar, with no statistically significant differences observed (all *P*>.05), as shown in Table 1.

**Table 1.** Demographic and health characteristics of the experimental and comparison groups.

Demographic and health characteristics	Experimental group (n=46)	Comparison group (n=45)		Statistical testing	
				Chi-square or *t* test (*df*)	*P* value
Sex, n (%)				0.4 (1)^a^	.55
Male	7 (15)	9 (20)			
Female	39 (85)	36 (80)			
Age (year)				1.504 (89)^b^	.14
Mean (SD; range)	68.57 (4.79; 60-78)	67.04 (4.85; 60-79)			
Age group, n (%)					
60‐65	12 (26)	19 (42)			
66‐70	15 (33)	15 (33)			
71‐75	19 (41)	11 (24)			
Religion, n (%)				3.2 (1)^a^	.07
Buddhism	42 (91)	35 (78)			
Islam	4 (9)	10 (22)			
Marital status, n (%)				0.009 (1)^a^	.93
Single, widowed, separated	26 (57)	25 (56)			
Couple	20 (43)	20 (44)			
Education, n (%)				1.9 (2)^a^	.38
Uneducated	2 (4)	5 (11)			
Primary or secondary	30 (65)	30 (67)			
Undergrade and graduate	14 (30)	10 (22)			
Occupation, n (%)				0.2 (2)^a^	.91
Unemployed	26 (56)	24 (53)			
Agriculture or merchant	11 (24)	11 (24)			
Retired	9 (20)	10 (22)			
Duration of hypertension				1.179 (89)^b^	.24
Mean (SD; range)	4.70 (0.80; 3-7)	4.51 (0.67; 4-7)			
Duration (years), n (%)					
Less than 5	30 (65)	34 (76)			
6‐10	16 (35)	11 (24)			

a Chi-square test.

b *t* test.

Most participants in both groups were female (n=39, 85% in the experimental group and n=36, 80% in the comparison group). The mean age was 68.57 (SD 4.79) years in the experimental group and 67.04 (SD 4.85) years in the comparison group. The majority of participants in both groups practiced Buddhism, followed by Islam. Regarding marital status, most participants were single, widowed, or separated (n=26, 57% in the experimental group and n=25, 56% in the comparison group). In terms of education, most had completed primary or secondary education (n=30, 65% in the experimental group and n=30, 67% in the comparison group). Regarding occupation, the majority in both groups were unemployed (n=26, 56% in the experimental group and n=24, 53% in the comparison group), with the remainder primarily engaged in agriculture or small trade.

### Effect of Health Monitoring Program on the Hypertension-Controlling Behavior

Before the intervention, there were no statistically significant differences between the experimental and comparison groups in overall or domain-specific hypertension-controlling behaviors, as shown in Table 2.

**Table 2.** Comparison of hypertension-controlling behaviors and mean arterial pressure of older adults with uncontrolled behavior between the experimental group and the comparison group.

Hypertension-controlling behaviors	Experimental group (n=46)			Comparison group (n=45)			*t* test (*df*)	*P* value
	Mean (SD)		Level	Mean (SD)		Level		
Food consumption								
Preprogram	3.27 (0.72)		Moderate	3.25 (0.69)		Moderate	0.101 (89)	.92
Postprogram	3.43 (0.66)		Moderate	3.41 (0.56)		Moderate	0.139 (89)	.89
Exercise								
Preprogram	3.38 (1.16)		Moderate	2.91 (1.21)		Moderate	1.891 (89)	.06
Postprogram	3.78 (0.90)		Very practiced	3.63 (1.13)		Very practiced	0.645 (89)	.52
Stress management								
Preprogram	3.76 (0.64)		Very practiced	3.62 (0.67)		Very practiced	0.992 (89)	.32
Postprogram	4.05 (0.56)		Very practiced	4.21 (0.62)		Very practiced	−1.196 (89)	.24
Medication adherence								
Preprogram	4.36 (0.66)		Very practiced	4.22 (0.68)		Very practiced	0.995 (89)	.32
Postprogram	4.49 (0.47)		Very practiced	4.46 (0.53)		Very practiced	0.281 (89)	.78
Hypertension-controlling behavior in total								
Preprogram	3.69 (0.49)		Very practiced	3.50 (0.50)		Moderate	1.832 (89)	.07
Postprogram	3.94 (0.42)		Very practiced	3.93 (0.50)		Very practiced	0.069 (89)	.94
Mean arterial pressure (mm Hg)								
Preprogram	107.14 (7.74)		High	106.86 (8.56)		High	0.163 (89)	.87
Postprogram	96.82 (8.85)		Normal	104.16 (10.52)		High	−3.604 (89)	<.001

After the 12-week program, the experimental group demonstrated significant within-group improvements, particularly in exercise and stress management domains (*P*<.05). Within-group analysis showed that the experimental group experienced a moderate improvement in hypertension-controlling behavior (Cohen *d*=0.55, 95% CI 0.24‐0.86), while the comparison group exhibited a large effect size (Cohen *d*=0.86, 95% CI 0.52‐1.20). However, despite the within-group improvements, no statistically significant difference was observed between groups in postprogram behavior scores, with a negligible effect size (Cohen *d*=0.02, 95% CI –0.39 to 0.43). This suggests that while the program may have supported individual improvement within the experimental group, its differential effect compared to usual care was limited (Table 3).

**Table 3.** Comparison of hypertension-controlling behaviors and mean arterial pressure of older adults with uncontrolled behavior between the pre- and postprogram enrolling.

Hypertension-controlling behaviors	Preprogram enrolling		Postprogram enrolling		*t* test	*P* value
	Mean (SD)	Level	Mean (SD)	Level		
Food consumption						
Experimental group	3.27 (0.72)	Moderate	3.43 (0.66)	Moderate	−1.242 (45)	.22
Comparison group	3.25 (0.69)	Moderate	3.41 (0.56)	Moderate	−1.720 (44)	.09
Exercise						
Experimental group	3.38 (1.16)	Moderate	3.78 (0.90)	Very practiced	−2.457 (45)	.02
Comparison group	2.91 (1.21)	Moderate	3.63 (1.13)	Very practiced	−4.584 (44)	<.001
Stress management						
Experimental group	3.76 (0.64)	Very practiced	4.05 (0.56)	Very practiced	−2.652 (45)	.01
Comparison group	3.62 (0.67)	Very practiced	4.21 (0.62)	Very practiced	−5.025 (44)	<.001
Medication adherence						
Experimental group	4.36 (0.66)	Very practiced	4.49 (0.47)	Very practiced	−1.420 (45)	.16
Comparison group	4.22 (0.68)	Very practiced	4.46 (0.53)	Very practiced	−2.586 (44)	.01
Hypertension-controlling behavior in total						
Experimental group	3.69 (0.49)	Very practiced	3.94 (0.42)	Very practiced	−3.202 (45)	.003
Comparison group	3.50 (0.50)	Moderate	3.93 (0.50)	Very practiced	−5.690 (44)	<.001
Mean arterial pressure (mm Hg)						
Experimental group	107.14 (7.74)	High	96.82 (8.85)	Normal	6.109 (45)	<.001
Comparison group	106.86 (8.56)	High	104.16 (10.52)	High	1723 (44)	.09

### Effect of Health Monitoring Program on the Mean Arterial Pressure

In the experimental group, SBP significantly decreased from 150.60 (SD 9.67) to 135.93 (SD 12.04) mm Hg, and DBP decreased from 85.41 (SD 9.30) to 77.26 (SD 8.59) mm Hg. The within-group effect size for SBP was a Cohen *d* of 1.34 (95% CI 0.94-1.74), and for DBP was a Cohen *d* of 0.91 (95% CI 0.57-1.25), indicating large effects [19,20]. MAP also showed a statistically significant reduction from 107.14 (SD 7.58) to 96.82 (SD 9.09) mm Hg, with a large effect size (Cohen *d*=1.22, 95% CI 0.83-1.60).

In contrast, the comparison group showed a nonsignificant reduction in SBP from 151.70 (SD 10.78) to 145.37 (SD 15.68) mm Hg, and a slight decrease in DBP from 84.44 (SD 9.83) to 83.56 (SD 10.08) mm Hg. The within-group effect size for SBP was a Cohen *d* of 0.47 (95% CI 0.16-0.78; moderate), and for DBP was a Cohen *d* of 0.09 (95% CI –0.20 to 0.38; negligible effect). MAP in the comparison group showed a minor, nonsignificant decrease from 106.86 (SD 8.69) to 104.16 (SD 10.62) mm Hg, with a small effect size (Cohen *d*=0.30, 95% CI –0.02 to 0.63).

## Discussion

### Principal Findings

#### Effect of Health Monitoring Program on the Hypertension-Controlling Behavior

The improved overall, exercise, and stress management behaviors in older adults with uncontrolled hypertension in the experimental group could be attributed to the program’s activities, which applied Bandura’s self-regulation concept through a 3-step process [17]. Self-observation involved older adults with uncontrolled hypertension using smartwatches to monitor key parameters like blood pressure, pulse, step count, and energy expenditure. A manual encouraged goal-setting to adjust these parameters, allowing them to track behaviors and performance outcomes to motivate behavior change and achieve their goals. The judgment *process* helped older adults compare their self-assessment results with personal goals using the “HT GeriCare@STOU” app and manual. It covered areas like diet, exercise, stress management, and medication adherence. Daily reflections allowed them to make adjustments and set new, personalized guidelines aligned with their goals. In self-reaction, older adults responded to their behavior evaluation from the judgment process. If they met their goals, they rewarded themselves positively; if not, they worked to improve and achieve better results.

While hypertension-controlling behaviors were high, they did not reach the highest level. Statistically significant improvements at the .05 level were observed in exercise and stress management. This improvement may be attributed to older adults in the experimental group, who tracked their daily activities using a smartwatch to count steps and assess pulse, ensuring exercise intensity aligned with their goals (55%‐80% of maximum pulse). In addition, videos sent via the LINE app in Weeks 2, 6, and 10 provided guidance on walking exercises and stress management techniques, such as relaxation breathing and positive thinking. While these interventions led to improved exercise and stress management behaviors, age-related limitations and some older adults’ independent lifestyles may have prevented them from achieving the highest level of performance.

Although food consumption and medication intake showed increased average values, they were not statistically significant at the .05 level. This may be due to limited ability to immediately change dietary habits, as meal preparation often depends on family caregivers. In addition, living in a large city like Bangkok, where ready-to-eat meals are widely available, older adults and their families often opted to buy food rather than cook, which made it harder to avoid high-fat foods, as reflected in the average scores on related questions.

The average scores for fruit and vegetable consumption among older adults in the experimental group were low, with about 2 handfuls consumed. This could be due to the availability of inexpensive, hard fruits like guavas and mangoes, which were difficult for older adults to chew. As for medication intake, older adults already displayed good adherence, so no significant changes were observed, especially in monitoring abnormal symptoms. Since they took their medication regularly, they tended to forget to observe these symptoms. However, older adults significantly demonstrated good dietary behaviors in reducing or avoiding sweet fruits and adding fewer seasonings to their meals. This knowledge was shared through videos in the LINE app during Weeks 2, 6, and 10, alongside food tracking in the “HT GeriCare@STOU” app. The food menu provided details on carbohydrates, proteins, fats, sugars, and sodium, helping older adults recognize the importance of various food types and assess their eating habits periodically, leading to positive behavioral changes in sweet and salty foods.

However, this study underscores the critical role of nurses as facilitators of technology-driven interventions. Nurses not only interpreted real-time health data but also provided individualized feedback, guided behavioral modifications, and supported adherence to hypertension management through telenursing home-based care. Their active involvement was pivotal in helping older adult participants understand their health status, translate data into actionable steps, and sustain hypertension-controlling behaviors. These findings highlight nurses’ unique capacity to bridge technology with patient-centered care, demonstrating their essential contribution to the successful implementation of mHealth interventions and reinforcing the broader implications for integrating such technologies into nursing practice and health systems.

#### Effect of Health Monitoring Program on the Mean Arterial Pressure

The improvement in MAP may be attributed to positive lifestyle behaviors promoted through the program, such as reduced intake of sweet fruits and excessive seasonings high in sugar and salt which could decrease blood vessel elasticity and narrow the arteries [21].

According to the Thai Hypertension Association (2019), reducing sodium to no more than 2300 milligrams per day could lower SBP by 2‐8 mm Hg [22], and further restriction to 1800 milligrams per day could lead to more substantial reduction by −5.0 mm Hg and DBP by −2.7 mm Hg in hypertensive patients [23].

Furthermore, participants in the experimental group showed significant improvements in stress management behaviors. Stress was a well-established contributor to hypertension through increased sympathetic activity and vascular resistance [24]. The program emphasized deep breathing exercises, which had been shown to increase vagal tone and reduce sympathetic dominance [25], thereby reducing blood pressure and stress [26,27]. In this study, systolic and diastolic pressures were significantly reduced by −14.67 mm Hg and −8.15 mm Hg, respectively, with large effect size (SBP: Cohen *d*=1.34, 95% CI 0.94‐1.74; DBP: Cohen *d*=0.91, 95% CI 0.57‐1.25) [19]. The impact of this program reduction exceeded that of home blood pressure monitoring alone [28], aligning with previous studies on self-monitoring combined with app-based feedback or mHealth interventions [29-32]. These results supported the assertion that the ontology framework used in this study functioned not merely as a classification system, but as an integrated decision-support tool. By linking symptom patterns and behavioral data to generate tailored feedback, it enabled personalized recommendations based on individual risk profiles and user behavior, ultimately enhancing participant engagement and improving self-care adherence.

The primary outcome of this study was the reduction in MAP, which showed significant improvement in the intervention group. Although overall behavioral scores did not differ significantly between groups, several specific lifestyle behaviors changed meaningfully. Participants in the intervention group reported reduced use of seasoning, decreased intake of sweetened foods, and increased daily step counts.

These targeted behavioral modifications, even if modest, likely contributed to the observed improvements in blood pressure. Consistent small changes in key behaviors can accumulate to produce meaningful physiological effects, even when overall behavioral scores appear unchanged. The combination of self-monitoring through wearable devices, real-time feedback, and personalized guidance from nurses likely reinforced these behaviors, enhancing adherence and translating into measurable MAP reductions.

This suggests that interventions focusing on key actionable behaviors, even without broad changes across all measured domains, can produce significant clinical benefits. Future studies could further explore how specific behavioral pathways mediate physiological outcomes and consider integrating objective behavioral metrics alongside self-reported measures to capture subtle but clinically relevant changes.

Feedback from older adult participants in the final evaluation indicated a high level of satisfaction (mean 4.16, SD 0.44). Similarly, nurse participants reported high satisfaction (mean 4.31, SD 0.41), noting that using the device allowed patients to observe daily results, increasing their awareness of blood pressure levels in relation to their recorded dietary intake. The nurses also observed that this real-time feedback appeared to motivate patients to independently improve their overall health.

While physiological mechanisms contributed to these improvements, practical factors also influenced the program’s impact. Several older adults faced challenges with device usability due to limited digital literacy, impaired vision, or hand tremors. Unstable internet connectivity in certain areas limited real-time interactions. Moreover, some participants experienced “tech fatigue” during extended screen use, which could hinder sustained engagement. These findings highlight the importance of ensuring that health technologies are user-friendly, accessible, and inclusive.

The use of ontology-based technologies and smart devices presented a promising approach for nurse practitioners to deliver care for hypertension [33]. However, the successful implementation of such systems depends on practical integration with clinical workflows. A recent systematic review noted that while artificial intelligence (AI) can enhance nursing productivity and allow more time for direct care, its effectiveness relied heavily on collaboration between system developers and practicing nurses [34]. Therefore, ongoing cooperation between technology designers and health care professionals is crucial to ensure usability and promote the adoption of digital health tools.

This study was conducted among urban older adults with access to smartphones, which may limit the generalizability of the findings to rural or low-resource settings. In areas with limited internet connectivity or lower digital literacy, participants may face difficulties using wearable devices and the ontology-based app. In addition, real-time follow-up and personalized guidance by nurses may be less feasible in remote locations due to resource constraints. Future studies should explore alternative approaches, such as low-tech interventions or telephone-based support, to adapt self-health monitoring programs for older adults in less-resourced contexts.

### Limitations

Due to information ethics, both older adults and the registered nurses were informed of the group to which they were assigned and the activities in which they were participating. As a result, older adults in the comparison group might have provided inaccurate information in their questionnaire responses, as they could have been influenced by the nurses caring for them. This awareness may have led to an unintentional increase in their hypertension-controlling behavior scores. The program’s applicability to older hypertensive patients with cellphones or a high education or economics—both of which are frequently found in big cities—is another drawback. Nonetheless, in crowded urban areas, the initiative helps shorten the time it took to go to patients’ houses.

Moreover, this study was conducted among older adults living in urban areas who had access to smartphones and internet connectivity. Therefore, these findings may not fully reflect the experiences of older adults in rural or low-resource settings, where digital literacy, technological access, and infrastructure may be limited. Future adaptations of the program should consider simplified interfaces, offline functionality, and involvement of community health volunteers to bridge the digital divide and improve accessibility for underserved populations.

Finally, from a methodological perspective, the use of *t* tests does not capture potential group×time interactions, which could provide deeper insights into behavioral changes over time. Future research with larger sample sizes should consider repeated measures ANOVA or mixed-effects models to more accurately evaluate longitudinal intervention effects.

### Conclusions

The self-health monitoring program—combining a smartwatch, the HT GeriCare@STOU app, telenursing through home-based care, and educational content via LINE—demonstrated effectiveness over a 12-week period. The program supported self-observation, self-assessment, and self-regulation, particularly enhancing stress management, exercise adherence, and modest dietary improvements.

Although most behavioral indicators did not differ significantly between groups, the experimental group showed a significant reduction in MAP. This suggests that physiological benefits may stem from increased self-awareness, psychological engagement, and stress reduction rather than overt behavior change. The motivational influence of technology and the psychological effect of being monitored may have further supported these outcomes.

These findings highlight the program’s utility as a motivational and supportive tool for hypertension self-care in older adults. Nurse practitioners could adopt this model to care for older adults with uncontrolled blood pressure, particularly those living in urban settings or with access to mobile technology.

## Supplementary material

10.2196/73386 Checklist 1 CONSORT-eHEALTH checklist (V 1.6.1).

## Abbreviations

2Q: 2-question
CONSORT: Consolidated Standards of Reporting Trials
DASH: dietary approaches to stop hypertension
DBP: diastolic blood pressure
MAP: mean arterial pressure
mHealth: mobile health
SBP: systolic blood pressure
